# Novel progesterone receptors: neural localization and possible functions

**DOI:** 10.3389/fnins.2013.00164

**Published:** 2013-09-19

**Authors:** Sandra L. Petersen, Karlie A. Intlekofer, Paula J. Moura-Conlon, Daniel N. Brewer, Javier Del Pino Sans, Justin A. Lopez

**Affiliations:** ^1^Molecular and Cellular Neuroendocrinology, Department of Veterinary and Animal Sciences, University of Massachusetts AmherstAmherst, MA, USA; ^2^Department of Psychology, University of VermontBurlington, VT, USA; ^3^Toxicology and Pharmacology Department, Complutense University of MadridMadrid, Spain; ^4^Department of Physiology and Pharmacology, Center for Neuroscience, West Virginia UniversityMorgantown, WV, USA

**Keywords:** PGR, PGRMC1, 25DX, PAQR, MPR

## Abstract

Progesterone (P_4_) regulates a wide range of neural functions and likely acts through multiple receptors. Over the past 30 years, most studies investigating neural effects of P_4_ focused on genomic and non-genomic actions of the classical progestin receptor (PGR). More recently the focus has widened to include two groups of non-classical P_4_ signaling molecules. Members of the Class II progestin and adipoQ receptor (PAQR) family are called membrane progestin receptors (mPRs) and include: mPRα (PAQR7), mPRβ (PAQR8), mPRγ (PAQR5), mPRδ (PAQR6), and mPRε (PAQR9). Members of the b5-like heme/steroid-binding protein family include progesterone receptor membrane component 1 (PGRMC1), PGRMC2, neudesin, and neuferricin. Results of our recent mapping studies show that members of the PGRMC1/S2R family, but not mPRs, are quite abundant in forebrain structures important for neuroendocrine regulation and other non-genomic effects of P_4_. Herein we describe the structures, neuroanatomical localization, and signaling mechanisms of these molecules. We also discuss possible roles for Pgrmc1/S2R in gonadotropin release, feminine sexual behaviors, fluid balance and neuroprotection, as well as catamenial epilepsy.

## Introduction

It is now clear that actions of progesterone (P_4_) in the nervous system go beyond its well-studied roles in regulating gonadotropin-releasing hormone (GnRH) release and feminine sexual behaviors (Chabbert-Buffeta et al., 2000; Mani and Blaustein, 2012). P_4_ also modulates such diverse processes as neuroprotection and neuroplasticity (Nilsen and Brinton, 2003; Peterson et al., 2012; Baudry et al., 2013; Sanchez et al., 2013), mood (Watson et al., 2012), neurogenesis (Bali et al., 2012) and neuroinflammation (Giatti et al., 2012). Therefore, it is not surprising that, in addition to the classical progestin receptor (PR), P_4_ exerts effects through multiple non-classical receptors.

Two groups of putative non-classical signaling molecules have emerged as likely mediators of P_4_ actions in the nervous system. One group consists of membrane P_4_ receptors (mPRs) that belong to the progestin and adipoQ receptor (PAQR) family. Five of these molecules, mPRα, mPRβ, mPRγ, mPRδ, and mPRε, are found in the brain (Thomas and Pang, 2012; Pang et al., 2013). These receptors contain seven trans-membrane domains and are thought to be unique G protein-coupled receptors that act through cAMP (Thomas and Pang, 2012). Of these receptors, mPRα, mPRβ, and mPRγ decrease cellular accumulation of cAMP, while mPRδ and mPRε increase accumulation (Karteris et al., 2006; Pang et al., 2013). There is evidence that mPRs are not always found in the plasma membrane or coupled to G proteins (Ashley et al., 2006; Krietsch et al., 2006; Smith et al., 2008). Thus, it has been suggested that they may function as alkaline ceramidases, enzymes that deacylate ceramides to produce lipid second messengers (Villa et al., 2009; Moussatche and Lyons, 2012). However, there is yet little data from mammalian cells to support this idea.

Members of a second group of molecules are structurally similar in that each contains a highly conserved cytochrome *b*5-heme/steroid binding domain (Kimura et al., 2012). This group includes progesterone receptor membrane component 1 [PGRMC1; also known as known 25Dx (Selmin et al., 1996)], PGRMC2, neudesin and neuferricin (Kimura et al., 2012). Each of these molecules is found in neural tissue (Krebs et al., 2000; Kimura et al., 2005, 2006, 2010; Intlekofer and Petersen, 2011), but only PGRMC1 has been reported to bind P_4_ (Meyer et al., 1996; Peluso et al., 2008, 2009). Recently, it has been suggested that PGRMC1 is the same molecule as the sigma-2 receptor (Xu et al., 2011). If this hypothesis is verified, it will expand our knowledge of how PGRMC1 might function in the nervous system because the sigma-2 receptor was primarily studied therein.

## Localization of P_4_ signaling molecules in specific nuclei of the brain

Although mPRs and PGRMC1-related molecules are found in the brain, most early studies did not compare the distributions of these molecules using techniques that provide detailed neuroanatomical information. Such information gives important clues to the functions regulated by the various receptors. Therefore, we used *in situ* hybridization (ISH) to map genes encoding PR, mPRα, mPRβ and PGRMC1, as well as its binding partners PGRMC2 and SERPINE 1 mRNA binding protein 1 (SERBP1), throughout the rat forebrain (Intlekofer and Petersen, 2011).

Somewhat surprisingly, neither *m*PR α nor *m*PR β is expressed specifically in neuroendocrine or other nuclei that mediate P_4_ functions (Intlekofer and Petersen, 2011). Moreover, except for very high mPRβ mRNA levels in the nucleus of the oculomotor cranial nerve, *m*PR α and *m*PR β expression is generally homogeneous and relatively low throughout the forebrain. In contrast, mRNAs encoding PGRMC1, PGRMC2 and SERBP1 are found in discrete neuroendocrine nuclei and in hippocampal, cortical and cerebellar regions that control functions modulated by P_4_ (Intlekofer and Petersen, 2011). More recently, we mapped expression of mPRδ and mPRε mRNAs in the rat forebrain and found no specific signal for either of these mRNAs (Moura-Conlon and Petersen, unpublished data).

**Figure 1:**
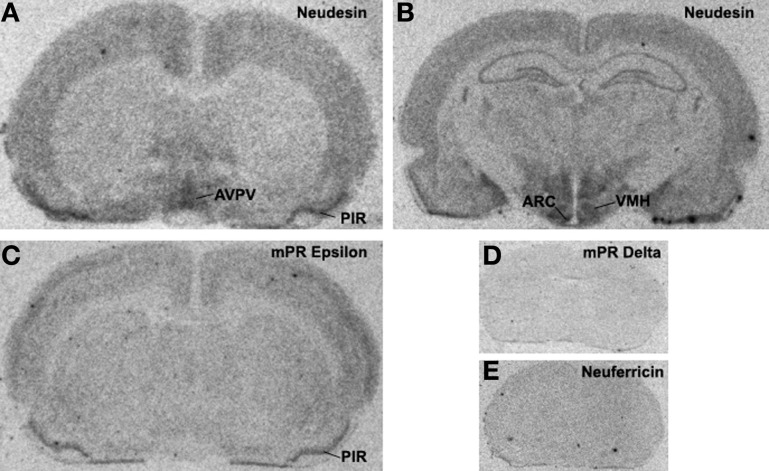
**X-ray film autoradiograms showing results of *in situ* hybridization studies.** Twelve-micron coronal frozen sections were hybridized to ^33^P-dATP end-labeled antisense deoxynucleotide probes for neudesin (Panels **A** and **B**), mPRε (Panel **C**), mPRδ (Panel **D**) or neuferricin (Panel **E**). Hybridization was as described previously (Intlekofer and Petersen, 2011) and sections were placed against Kodak BioMax MR film (Rochester, NY) for 2 weeks.

Neuferricin is a recently discovered extracellular heme-binding protein that facilitates neurogenesis in cultured progenitor cells (Kimura et al., 2010). In preliminary *in situ* hybridization studies, we failed to detect neuferricin mRNA in the rat forebrain. In contrast, the distribution pattern of *neudesin* gene expression is strikingly similar to that of *pr* in the rat forebrain, particularly in regions containing the anteroventral periventricular, arcuate, and the ventromedial nuclei [compare Figure 1 and (Simerly et al., 1996; Shughrue et al., 1997)]. This 171-amino acid secreted protein is expressed in neural, but not glial cells (Kimura et al., 2005). Similarly, it promotes differentiation of neurons and inhibits differentiation of astrocytes (Kimura et al., 2006). Neudesin exerts these effects through protein kinase and phosphatidylinositol-3 kinase pathways (Kimura et al., 2006), and its cytochrome *b*_5_-like heme/steroid-binding domain is also required (Kimura et al., 2008). The role of neudesin in the regulation of adult neural functions is unclear, but the striking similarity of the neudesin and PR mRNA distribution patterns (Figure 1) suggests the possibility that neudesin may act in concert with PR to regulate neuroendocrine functions.

Our neuroanatomical findings indicate that *pgrmc1* is the most abundant putative membrane P_4_ receptor gene expressed in neuroendocrine regions; therefore, this review focuses on possible roles of PGRMC1 in regulating some of these functions. For a more detailed review of all the putative non-classical P_4_ signaling molecules, see (Petersen et al., 2013).

## Structures of PGRMC1 and PGRMC2

PGRMC1 has been partially purified from liver membranes (Meyer et al., 1996), spontaneously immortalized rat granulosa cells (Peluso et al., 2008), and human granulosa/luteal cells (Peluso et al., 2009). Results of studies using these preparations suggests that PGRMC1 binds P_4_ with high affinity [k_d_ estimates of 10, 11, and 35 nM (Meyer et al., 1996; Peluso et al., 2008, 2009)]. However, the idea that PGRMC1 alone binds P_4_ is not universally accepted (Rohe et al., 2009). For example, Min *et al*. found that GST-tagged rat inner zone antigen [found to be identical to PGRMC1; see (Cahill, 2007)] expressed in *E. coli* did not bind P_4_ in pull-down assays (Min et al., 2005).

It is possible that there are other P_4_-binding proteins in the partially purified preparations wherein binding has been detected (Meyer et al., 1996; Peluso et al., 2008, 2009), but studies in rat granulosa cells suggest that PGRMC1 accounts for the specific P_4_ binding. Peluso and colleagues showed that partially purified GFP-PGRMC1 fusion protein binds P_4_ with nM affinity and deletions in various parts of the PGRMC1 molecule reduce P_4_ binding (Peluso et al., 2008). SERBP1 (also called plasminogen activator inhibitor 1 RNA binding protein; PAIRBP1) is important for PGRMC1 functions (Peluso et al., 2013), but the P_4_ binding site on PGRMC1 and the site for SERBP1 interaction differ (Peluso et al., 2008). In addition, depletion of SERBP1 increases, rather than decreases, P_4_ binding in spontaneously immortalized granulosa cells (Peluso et al., 2013). Finally, perhaps the most compelling evidence that PGRMC1 binds P_4_ comes from work showing that knockdown of PGRMC1 by 60% reduces P_4_ binding by the same percentage (Peluso et al., 2008).

Few studies have examined binding of steroids other than P_4_ to PGRMC1. Early work characterizing PGRMC1showed that P_4_, but not dexamethasone, aldosterone or β-estradiol bind specifically to partially purified PGRMC1 in microsomal or solubilized membrane fractions from porcine liver (Meyer et al., 1996). In the same studies, it was found that corticosterone and testosterone bind with affinities ~25% that of P_4_, and cortisol with a relative affinity of 4%. Thus, PGRMC1 appears to preferentially bind P_4_.

PGRMC1 is relatively small [194 amino acids (Falkenstein et al., 1996)] with a molecular weight estimated between 25 and 28kDa (Meyer et al., 1996; Selmin et al., 1996; Raza et al., 2001; Peluso et al., 2009). However, higher molecular weight molecules can also be detected and likely represent dimers (Meyer et al., 1996), multimers (Losel et al., 2005), or molecules modified post-translationally through sumoylation or other processes (Peluso et al., 2012). PGRMC1 contains an N-terminal extracellular domain, a transmembrane domain, and a cytoplasmic region with a heme-binding domain (Peluso et al., 2006; Cahill, 2007).

Consistent with evidence that PGRMC1 and sigma-2 receptors are the same protein (Xu et al., 2011), the two molecules have similar steroid hormone-binding profiles with high affinity for P_4_ (Meyer et al., 1996; Peluso et al., 2008). Moreover, sigma-2 ligand binding colocalizes with PGRMC1 expression in the endoplasmic reticulum (ER) and mitochondria (Xu et al., 2011). In addition, changes in PGRMC1levels correlate with changes in sigma-2 ligand binding (Xu et al., 2011). Finally, sigma-2 receptors regulate intracellular calcium levels (Vilner and Bowen, 2000) and apoptosis (Vilner and Bowen, 1993; Vilner et al., 1995), as does PGRMC1 (Viero et al., 2006; Peluso et al., 2008; Bashour and Wray, 2012; Lai et al., 2012).

PGRMC2 is structurally similar to PGRMC1 (Cahill, 2007; Wendler and Wehling, 2013), except in the N-terminus and transmembrane domain. This finding may explain why PGRMC2 does not bind P_4_ (Peluso, Pers. Commun.), because the P_4_ binding site of PGRMC1 is in the transmembrane domain [26]. In contrast, PGRMC1 and PGRMC2 both bind the same members of a group of heme-containing molecules, the cytochrome P450 proteins (Albrecht et al., 2012), suggesting that the heme-binding sites function similarly in PGRMC1 and PGRMC2. It is notable that PGRMC2 expression widely overlaps that of PGRMC1 in brain nuclei (Intlekofer and Petersen, 2011). However, the role of PGRMC2 in P_4_ signaling in the nervous system or in other tissues remains unclear.

## Possible roles for PGRMC1 in regulating rapid neuroendocrine responses

### Gonadotropin release

Most studies examining PGRMC1 functions have focused on non-neural reproductive tissues such as the ovary (Kowalik and Kotwica, 2008; Peluso, 2011) and uterus (Zhang et al., 2008). Results of our neuroanatomical studies suggest that PGRMC1 and its partners also regulate the neural structures that control reproductive functions. The region in which mRNAs encoding PGRMC1, PGRMC2 and SERBP1 are highest is the anteroventral periventricular nucleus (AVPV), a group of cells in which E_2_ acts to induce luteinizing hormone (LH) surge release and ovulation in rodents (Petersen et al., 2003). E_2_ triggers the LH surge mechanism, at least in part, by upregulating PR expression in the AVPV (Chappell and Levine, 2000) and by increasing synthesis of neurosteroids in the region (Micevych and Sinchak, 2008, 2011). The surge is then rapidly amplified by rising levels of circulating P_4_ (Levine, 1997). Based on the high levels of expression in the AVPV, it is possible that PGRMC1 may mediate one or both of these rapid effects of P_4_. Unfortunately, this idea is difficult to test because the LH surge does not occur in the absence of PR (Chappell et al., 1997, 1999).

One possible way in which PGRMC1 might enhance LH surge release is by increasing neurosteroid synthesis in the AVPV. Local steroid production in the AVPV is important for the LH surge (Micevych and Sinchak, 2011) and PGRMC1 enhances the activity of cytochrome P450 (Cyp) enzymes involved in steroid synthesis (Hughes et al., 2007; Rohe et al., 2009; Ahmed et al., 2012). For example, through its heme-binding site, PGRMC1 binds to and enhances the activity of Cyp51, an enzyme necessary for the conversion of lanosterol to cholesterol (Craven et al., 2007; Hughes et al., 2007). This is a key finding because cholesterol does not appear to be synthesized in the brain (Bjorkhem and Meaney, 2004). Similarly, PGRMC1 activates Cyp19 aromatase, an enzyme necessary for E_2_ synthesis (Ahmed et al., 2012), a hormone that acts in the AVPV to induce the LH surge (Petersen et al., 2003). It has not yet been determined whether PGRMC1 regulates the activity of other heme-dependent Cyp enzymes involved in P_4_ synthesis or its metabolism to other neuroactive progestins such as allopregnanolone. However, it seems likely considering that PGRMC1 regulates nearly all Cyp enzymes tested to date (Ahmed et al., 2012). Thus, PGRMC1 may indirectly amplify the preovulatory LH surge by enhancing activity of enzymes involved in neurosteroid synthesis and metabolism.

It is also possible that PGRMC1 mediates rapid inhibitory effects of P_4_ on LH release. Both PGRMC1 and SERBP1 are detected in nearly all GnRH neurons of embryonic explants, (Bashour and Wray, 2012). Similarly, PGRMC1 is found in immortalized GnRH neurons, GT1-7 cells (Krebs et al., 2000). As in non-neural cells (Peluso et al., 2002), P_4_ rapidly inhibits fluctuations in intracellular calcium levels in GnRH neurons through mechanisms that do not involve GABA_A_ receptors (Bashour and Wray, 2012) as have been described previously in embryonic sensory neurons (Viero et al., 2006). Rather, a putative PGRMC1 antagonist blocks the inhibitory effect of P_4_ and, consistent with evidence that PGRMC1 signals through PKG (Peluso and Pappalardo, 2004; Peluso et al., 2007), PKG inhibitors block P_4_ inhibition of calcium flux in explant GnRH neurons (Bashour and Wray, 2012). Thus, PGRMC1 may be important for turning off the LH surge or limiting it to one day of the cycle.

**Figure 2:**
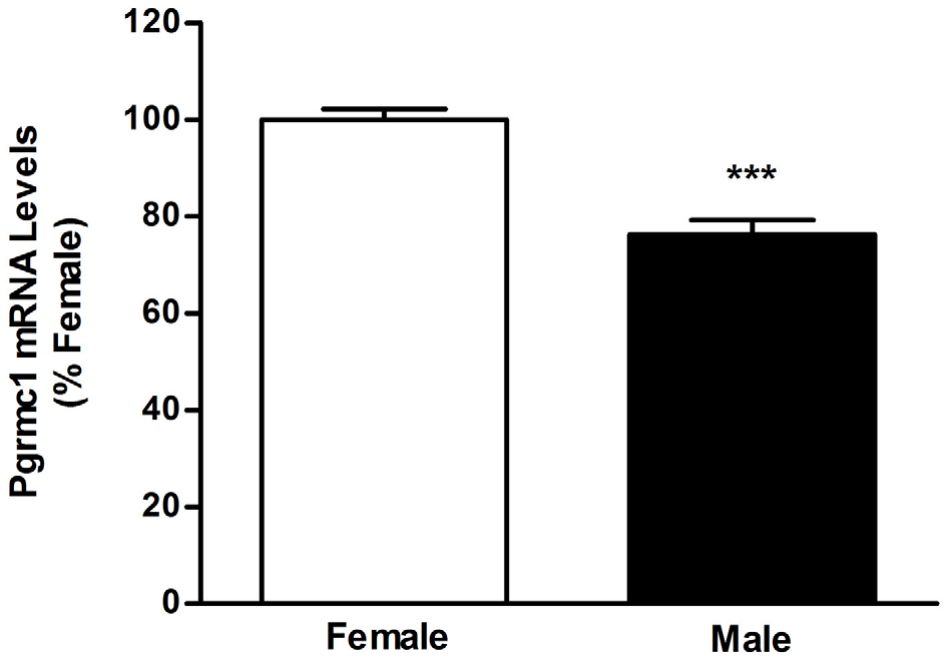
**Results of quantitative real-time polymerase chain reaction analyses measuring PGRMC1 mRNA in microdissections of postnatal day 2 female and male anteroventral periventricular nuclei.** ^***^Significantly different from female; *p* < 0.0001.

PGRMC1 is also interesting in the context of sexual differentiation of brain nuclei, particularly of preoptic area and hypothalamic nuclei that develop through sex-specific and E_2_-regulated apoptosis. Sexual differentiation of the AVPV occurs during the perinatal period when the developing testes, but not ovaries, are active. In the male AVPV, testosterone is aromatized to E_2_ and this hormone triggers apoptosis (Arai et al., 1996; Forger, 2009; Tsukahara, 2009) and defeminization of LH release mechanisms. Importantly, PGRMC1 prevents apoptosis in non-neural tissue (Peluso et al., 2009) and we recently found that PGRMC1 mRNA levels are lower in the neonatal AVPV of males than females (Figure 2). Moreover, the arylhydrocarbon receptor ligand, 2,3,7,8-tetrachlorodibenzo-*p*-dioxin, increases *pgrmc1* gene expression (Selmin et al., 1996) and developmental exposure to TCDD blocks defeminization of LH release (Mably et al., 1992). Thus, indirect evidence suggests that PGRMC1 may prevent cell death in the developing AVPV.

### Feminine sexual behaviors

In addition to its effects on GnRH and LH release, P_4_ also rapidly enhances female mating behaviors through actions in brain regions that contain PGRMC1. Most of these brain regions also contain dopamine (DA) receptors and PGRMC1/sigma-2R agonists increase DA release (Garcés-Ramírez et al., 2011). For example PGRMC1/sigma-2R mRNA levels are high in the medial preoptic area (Krebs et al., 2000; Intlekofer and Petersen, 2011), a region in which P_4_ increases DA release (Matuszewich et al., 2000). DA, in turn, acts through DA D2 receptors in the medial preoptic area to enhance feminine precopulatory behaviors (Graham and Pfaus, 2010). In addition, DA input into the medial preoptic area comes primarily from the zona incerta (Wagner et al., 1995), a region that contains high levels of PGRMC1, PGRMC2, and SERBP1mRNA. Levels are also high in the ventromedial hypothalamus (Krebs et al., 2000; Intlekofer and Petersen, 2011), a brain region in which both P_4_ (Flanagan-Cato, 2011) and DA D1 and D5 receptor agonists induce lordosis (Apostolakis et al., 1996). Finally, expression of PGRMC1 and binding partners is prominent in the ventral tegmental area (VTA) wherein P_4_ regulates the maintenance of lordosis through actions involving DA D1 and D5 receptors(Sumida et al., 2005).

As is the case with GnRH/LH surge release, PR is required for the appearance of female mating behaviors, and both classical and ligand-independent activation of PR play a role (Mani and Blaustein, 2012). In fact, at least some of the DA-mediated effects on behavior require PR (Mani et al., 2009; Mani and Blaustein, 2012); however, that does not preclude the possibility that PGRMC1 also participates in the process. For example, sigma-2 ligands regulate DA transporter activity through a Ca^2+^/calmodulin-dependent protein kinase II (CaMKII) transduction system (Weatherspoon and Werling, 1999). Likewise, P_4_ effects on female mating behavior involve rapid changes in CaMKII kinase activity in the ventromedial hypothalamus (Balasubramanian et al., 2008) through a mechanism upstream from DA receptor activation (Balasubramanian and Mani, 2009). Thus, it may be that P_4_ regulates DA release, reuptake and binding, as well as downstream signaling through a combination of classical and non-classical P_4_ receptors.

PGRMC1 may also affect DA signaling and female reproductive behaviors by altering NMDA-type glutamate receptor functions. As described above, the VTA is a site in which P_4_ facilitates female sexual behaviors (Debold and Malsbury, 1989). Therefore, it is intriguing that sigma-2 receptor agonists, which presumably bind PGRMC1 (Xu et al., 2011), potentiate NMDA-induced activity of DA neurons in the VTA (Gronier and Debonnel, 1999). Considering that DA signaling, but not PR action, in the VTA is required for lordosis (Frye and Vongher, 1999; Sumida et al., 2005), these findings support the idea that PGRMC1 plays a role. However, this is likely to be a complicated story because NMDA signaling in the VTA suppresses lordosis quotients under some circumstances (Frye et al., 2008). Thus, if PGRMC1 is part of the signaling mechanism that controls lordosis, it may be involved in turning off the behavior. Alternatively, it may be that responses to glutamate depend on the particular subregion of the VTA affected.

### Other potential neural functions of PGRMC1

Results of our neuroanatomical studies suggest that PGRMC1 may also mediate effects of P_4_ on non-reproductive functions. Two of the regions in which PGRMC1 was first detected are the supraoptic and paraventricular nuclei (Krebs et al., 2000; Meffre et al., 2005). These regions contain among the highest levels of PGRMC1, PGRMC2, and SERBP1 mRNA in the rat forebrain (Intlekofer and Petersen, 2011). PGRMC1is also expressed in circumventricular organs, ependymal cells and meninges, and colocalizes with vasopressin in paraventricular and supraoptic nuclei; therefore, it has been proposed that PGRMC1 might be involved in water homeostasis in the brain (Meffre et al., 2005). Support for this idea comes from evidence that PGRMC1 expression increases in neurons and appears in astrocytes after traumatic brain injury that results in edema (Meffre et al., 2005). This finding has important clinical implications because P_4_ is now in clinical trials to evaluate its effectiveness on decreasing brain damage in stroke and traumatic brain injury (Stein, 2011). It remains to be determined whether P_4_ acts, at least in part, through PGRMC1 to exert its neuroprotective effects by controlling edema in the brain.

Finally, the piriform cortex is a part of the limbic system and both PGRMC1 and SERBP1 mRNA levels are very high in this region, while PR and mPR mRNAs are quite low or absent (Intlekofer and Petersen, 2011). This is not a well-studied brain structure from a neuroendocrine perspective, but the piriform cortex is central to the development and propagation of kindled seizures (Wahnschaffe et al., 1993; Loscher et al., 1995). Although no changes in kindled seizure threshold are observed during the estrous cycle of rats (Wahnschaffe and Loscher, 1992), many women with epilepsy experience seizures clustered around the time of the menstrual cycle when P_4_ levels are low (termed catamenial epilepsy) (Duncan et al., 1993; Herzog et al., 2004; Gilad et al., 2008; Reddy, 2009). P_4_ can significantly reduce the frequency of seizures in women with this disease (Reddy, 2009; Motta et al., 2013). Further studies are required to determine whether PGRMC1 plays a role in catamenial epilepsy and whether PGRMC1/sigma-2 ligands would be effective in treating the disease without the side effects of progestins.

## Summary

A large body of literature catalogues the many neural actions of PR accomplished through gene regulation or rapid regulation of intracellular signaling. In contrast, there is relatively little work on the role of PGRMC1 or other non-classical P_4_ signaling molecules in the brain. Based on our neuroanatomical findings that PGRMC1, PGRMC2, and SERBP1 are found in brain regions wherein P_4_ exerts rapid effects, it seems likely that these molecules are involved in diverse functions. These functions include the control of GnRH/LH release, feminine mating behaviors, fluid balance, and neuroprotection and seizure activity. The extensive overlap in patterns and levels of expression suggest that PGRMC1 and PR signaling pathways regulate the same cellular functions, but probably through different mechanisms. Considering the importance of these functions in physiology and disease, further study of PGRMC1, PGRMC2, and SERBP1 in the nervous system is warranted.

### Conflict of interest statement

The authors declare that the research was conducted in the absence of any commercial or financial relationships that could be construed as a potential conflict of interest.
